# Emerging Applications of Bedside 3D Printing in Plastic Surgery

**DOI:** 10.3389/fsurg.2015.00025

**Published:** 2015-06-16

**Authors:** Michael P. Chae, Warren M. Rozen, Paul G. McMenamin, Michael W. Findlay, Robert T. Spychal, David J. Hunter-Smith

**Affiliations:** ^1^3D PRINT Laboratory, Department of Surgery, Peninsula Health, Frankston, VIC, Australia; ^2^Monash University Plastic and Reconstructive Surgery Group (Peninsula Clinical School), Peninsula Health, Frankston, VIC, Australia; ^3^Department of Anatomy and Developmental Biology, Centre for Human Anatomy Education, School of Biomedical Sciences, Faculty of Medicine, Nursing and Health Sciences, Monash University, Clayton, VIC, Australia; ^4^Department of Surgery, Stanford University, Stanford, CA, USA

**Keywords:** 3D printing, bedside, desktop application, plastic and reconstructive surgery, cost, preoperative planning, intraoperative guidance, education

## Abstract

Modern imaging techniques are an essential component of preoperative planning in plastic and reconstructive surgery. However, conventional modalities, including three-dimensional (3D) reconstructions, are limited by their representation on 2D workstations. 3D printing, also known as rapid prototyping or additive manufacturing, was once the province of industry to fabricate models from a computer-aided design (CAD) in a layer-by-layer manner. The early adopters in clinical practice have embraced the medical imaging-guided 3D-printed biomodels for their ability to provide tactile feedback and a superior appreciation of visuospatial relationship between anatomical structures. With increasing accessibility, investigators are able to convert standard imaging data into a CAD file using various 3D reconstruction softwares and ultimately fabricate 3D models using 3D printing techniques, such as stereolithography, multijet modeling, selective laser sintering, binder jet technique, and fused deposition modeling. However, many clinicians have questioned whether the cost-to-benefit ratio justifies its ongoing use. The cost and size of 3D printers have rapidly decreased over the past decade in parallel with the expiration of key 3D printing patents. Significant improvements in clinical imaging and user-friendly 3D software have permitted computer-aided 3D modeling of anatomical structures and implants without outsourcing in many cases. These developments offer immense potential for the application of 3D printing at the bedside for a variety of clinical applications. In this review, existing uses of 3D printing in plastic surgery practice spanning the spectrum from templates for facial transplantation surgery through to the formation of bespoke craniofacial implants to optimize post-operative esthetics are described. Furthermore, we discuss the potential of 3D printing to become an essential office-based tool in plastic surgery to assist in preoperative planning, developing intraoperative guidance tools, teaching patients and surgical trainees, and producing patient-specific prosthetics in everyday surgical practice.

## Introduction

Advanced medical imaging has become an essential component of preoperative planning in plastic surgery. In breast reconstructive surgery, the introduction of computed tomographic angiography (CTA) has enabled surgeons to improve clinical outcomes (1) through accurate and reliable prospective selection of the donor site, flap, perforators, and the optimal mode of dissection (2, 3). Recent development of three-dimensional (3D) and 4D CTA techniques have enhanced spatial appreciation of the perforator vessels, their vascular territory, and dynamic flow characteristics preoperatively (4, 5). However, current imaging modalities are limited by being displayed on a 2D surface, such as a computer screen. In contrast, a 3D-printed haptic biomodel allows both the surgeon and the patient to develop a superior understanding of the anatomy and the procedure with the goal of improved operative planning through the ability to interact directly with a model of the patient-specific anatomy. Historically, the technically challenging nature of 3D software and the high prices of early 3D printers usually meant that clinicians keen to exploit these advantages had to outsource 3D printing and the cost of outsourcing often precluded it from being implemented widely. In this review, we analyze how recent advancements have enabled 3D printing to transition from the research and development laboratory to the clinical ‘bedside’ potentially making it a ubiquitous application in plastic surgery.

## 3D Printing

**Figure 1:**
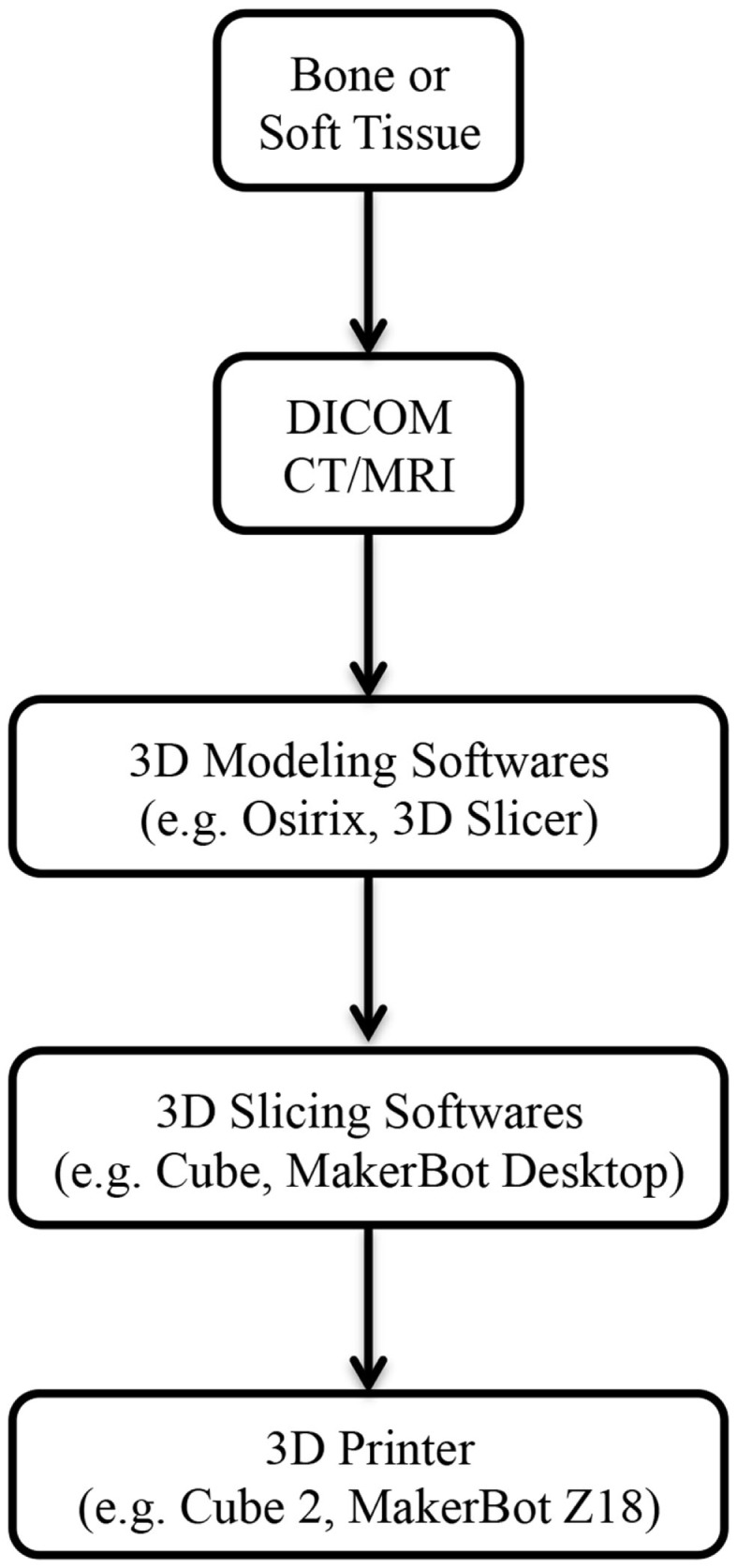
**Steps involved from imaging to 3D-printed models**. Abbreviations: DICOM, digital imaging and communications in medicine; CT, computed tomography; MRI, magnetic resonance imaging.

3D printing, also known as rapid prototyping or additive manufacturing, describes a process by which a product derived from a computer-aided design (CAD) is built in a layer-by-layer fashion (Figure 1) (Video S1 in Supplementary Material) (6–8). In contrast to the conventional manufacturing processes like injection molding, 3D printing has introduced an era of design freedom and enabled rapid production of customized objects with complex geometries (9–11). One of the major advantages of 3D printing is the capacity to directly translate a concept into an end product in a convenient, cost-efficient manner. It eliminates the typical intermediary stages involved in a product development, such as development, production, assembly lines, delivery, and warehousing of parts (12), and the subsequent savings made from using fewer materials and labor lead to an overall reduction in the cost of production (13).

3D printing has been utilized in industrial design since the 1980s; however, it has only become adapted for medical application in the last decade (14). Imaging data from routine computed tomography (CT) or magnetic resonance imaging (MRI) can be converted into a CAD file using a variety of 3D software programs, such as Osirix (Pixmeo, Geneva, Switzerland) or 3D Slicer (Surgical Planning Laboratory, Boston, MA, USA) (Figure 1). These files are processed into data slices suitable for printing by proprietary softwares from the 3D printer manufacturers. While a range of 3D printing techniques have been developed for industrial use; stereolithography (SLA), multijet modeling (MJM), selective laser sintering (SLS), binder jetting, and fused deposition modeling (FDM) are the main approaches that have been explored in the clinical setting (Table 1). We will explore each of these to evaluate their current and potential applications in clinical practice for both bony reconstruction and soft tissue reconstruction.

**Table 1:** **A summary of the most commonly used 3D printing techniques in medical application**.

3D printing techniques	Pros	Cons
SLA	Current gold standard High resolution Increased efficiency with increase in print size Detailed fabrication of internal structures	>1 day of printing time required Require extensive post-production manual handling High cost related to the materials, the printer, and the maintenance
MJM	High resolution Minimal post-production manual handling Multiple materials	High cost related to the material and printer Poorer surface finishing than SLA
SLS	Not require support structures Smooth surface finishing Print delicate structures Print in metal	Require post-production manual handling High cost related to the materials, the printer, and the maintenance Require expert handling of the printer
BJT	Not require support structures Multiple colors Multiple materials	Brittle Require extensive post-production manual handling Poor surface finish
FDM	Low cost Minimal maintenance High availability of printers	Require post-production manual removal of support structures Poor surface finish Mono-color and mono-material with the current technology

*SLA, stereolithography; MJM, multijet modeling; SLS, selective laser sintering; BJT, binder jet technique; FDM, fused deposition modeling*.

### Types of 3D printing

#### Stereolithography

Stereolithography is the earliest 3D printing technology described for fabricating biomodels, where a layer of liquid photopolymer or epoxy resin in a vat is cured by a low-power ultraviolet (UV) laser (15). Excess raw materials and the supporting structures must be manually removed from the final product and cured in a UV chamber (16–18). Currently, SLA is considered the gold standard in 3D biomodel production and can yield resolutions of up to 0.025 mm. Moreover, its efficiency increases when constructing larger objects and is able to faithfully reproduce internal structural details (19). However, the need for manual post-build handling makes it labor-intensive and it still takes more than a day to produce a large model. Furthermore, in comparison to other 3D printing techniques, it is considered more expensive due to the high cost of the raw materials and for the printer upkeep (20, 21). Recently, a novel modification to SLA has been developed called continuous liquid interface production (CLIP). This simplifies traditional SLA and increases the production speed by harnessing oxygen inhibition of UV-curable resin photopolymerization (22). This emerging modality has yet to be evaluated in plastic and reconstructive surgery but holds promise due to its combination of speed, structural integrity, and ability to fabricate complex structures.

#### MultiJet Modeling

Multijet modeling printing, also known as MultiJet Printing (3D Systems, Rock Hill, SC, USA) or Poly Jet Technology (Stratasys, Edina, MN, USA), is akin to SLA, but the liquid photopolymer is immediately cured by the UV light preventing the time-consuming post-processing in the UV chamber and the prototypes are built with gel-like support materials that are readily dissolvable in water (23). MJM can manufacture models with high resolution (16 μ) that is comparable to or better than SLA, with an added benefit of the capacity to print in multiple materials for the desired degree of tensile strength and durability. Furthermore, a MJM printer is easier to maintain than a SLA set-up. However, the high price of these printers makes MJM more suitable for large-scale productions than for office-based/bedside desktop application.

#### Selective Laser Sintering

Selective laser sintering describes a process where powdered forms of thermoplastic, metal, glass, or ceramic material are sintered by high-power laser beams in a layer-by-layer fashion (24, 25). Similar to SLA, the unsintered powders must be brushed away from the final product; however, they provide support and eliminate the need for support structures. As a result, SLS yields models with smoother surface finish and facilitates the production of delicate structures with high accuracy. Furthermore, the unsintered powders can be reused leading to a reduction in cost compared to SLA (20, 26). However, SLS remains significantly more expensive than binder jet technique (BJT) (below) and FDM, due mainly to the cost of the printer. In addition, SLS printers can be potentially hazardous due to the presence of lasers, pistons, and gas chambers that can reach extremely high temperatures and hence, requires expert handling. These features have discouraged it from being widely implemented in non-industrial settings.

#### Binder Jet Technique

Binder jet technique, or powder bed technique, is the first 3D printing approach that reduced the cost of 3D printers, thereby enabling a widespread consumerization of 3D printing (27). Similar to the SLS process, printer heads eject a binder material along with colored dye onto a layer of powder, fusing them layer-by-layer into a plaster model (28). Unfused powders provide adequate support for the “overhanging” designs and hence, simultaneous deposition of support structures is rarely required. Moreover, binder jet 3D printers can print in multiple colors and materials, and have multiple printer heads for faster printing. One of the major drawbacks of binder jetting is that the final product usually lacks strength and has a poorer surface finish than SLA or SLS. Hence, all models require post-production strengthening with materials such as melted wax, cyanoacrylate glue, or epoxy.

#### Fused Deposition Modeling

Fused deposition modeling is the most commonly used consumer 3D printing technology available currently and is also the most affordable (21, 29, 30). A melted filament of thermoplastic material is extruded from a nozzle moving in the x-y plane and solidifies upon deposition on a build plate (31). After each layer, the build plate is lowered by 0.1 mm and the process is repeated until the final product is produced. Acrylonitrile-butadiene-styrene (ABS) and polylactic acid (PLA) are the most frequently used raw materials in FDM printers. A notable shortcoming for the use of FDM in medical applications is that most anatomical structures have complex shapes and hence, would require support structures. Although they are easy to remove manually, the aftermath generally leaves superficial damage to the model compromising its surface finish and esthetics. Hollow internal structures or blind-ended openings are particularly difficult to clean build material from. Furthermore, most household FDM printers are currently limited to fabricating in mono-color and mono-material. However, this can be overcome by recently developed dual-extruder technology, where two filaments of different color or material can be extruded from a common printer head. It is currently found in printers, such as MakerBot Replicator 2X Experimental (MakerBot Industries, New York, NY, USA), Cube 3 (3D Systems, Rock Hill, SC, USA), and Creatr x1 (Leapfrog, Emeryville, CA, USA). Moreover, the second extruder can be configured to build support structures using MakerBot Dissolvable Filament (MakerBot Industries), made up of high impact polystyrene (HIPS) (32). When the final product is immersed in water with limonene, a widely available citrus-scented solvent, the support structures selectively dissolve away within 8 to 24 h but these dual extruder printers have not yet become established in the mainstream.

## 3D Printing in Medicine

In the last decade or so, researchers have demonstrated a wide range of uses for 3D printing across numerous surgical disciplines. Clinically, 3D-printed haptic biomodels provide a tactile feedback and enable users to simulate complex anatomical movements, such as articulation at the temporomandibular joint, that are difficult to reproduce in a computer software (33). As a result, they facilitate an enhanced appreciation of the visuospatial relationship between anatomical structures for the surgeons (34). This can translate into shorter operative time, reduced exposure to general anesthesia, shorter wound exposure time and reduced intraoperative blood loss (18, 35, 36).

### Preoperative planning

**Figure 2:**
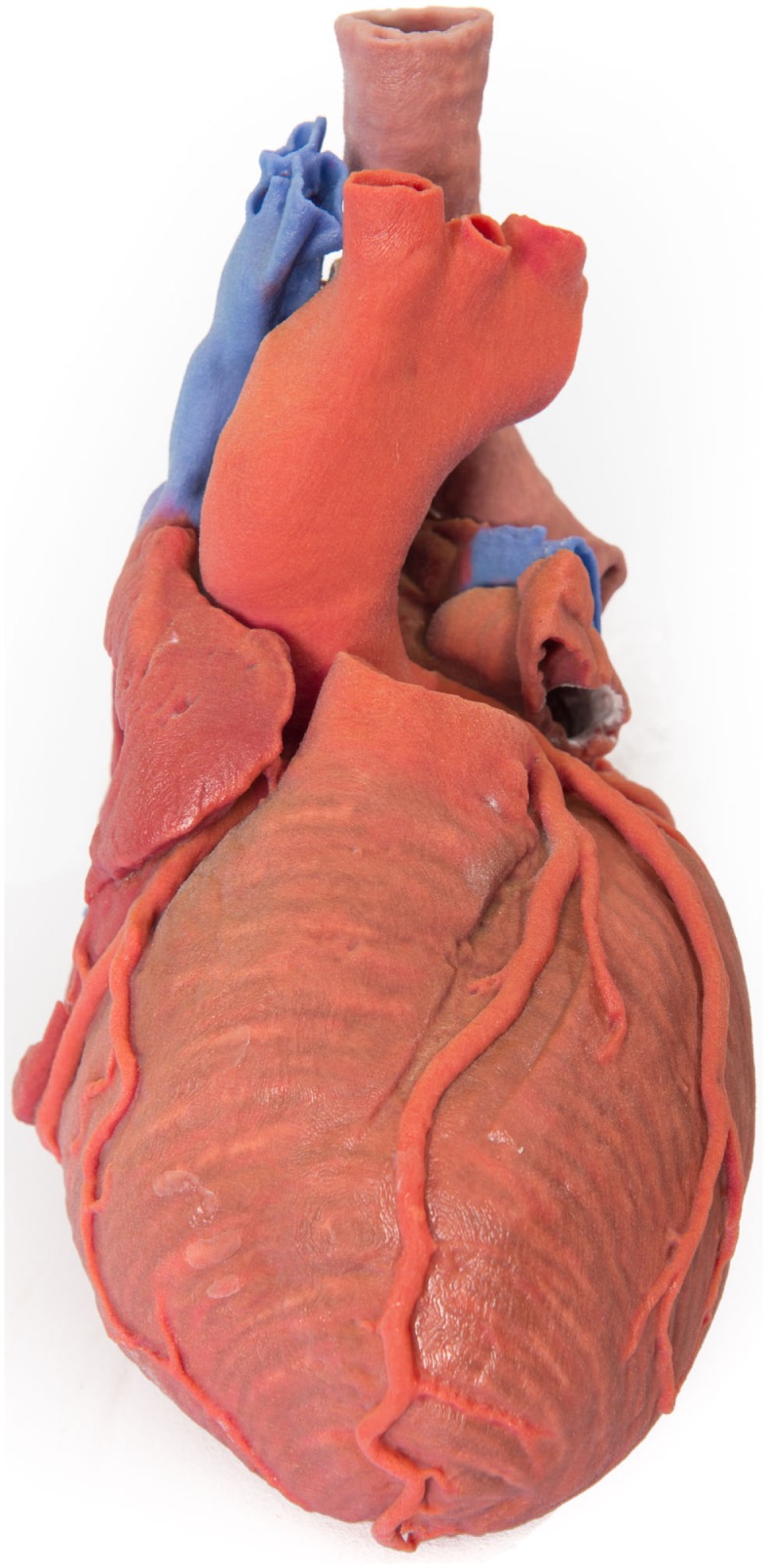
**3D-printed haptic model of a heart and the great vessels fabricated using Projet x60 series 3D printers**. Reproduced with permission from Centre for Human Anatomy and Education.

In preoperative planning, 3D-printed biomodels have been beneficial in orbital and mandibular reconstruction in maxillofacial surgery (21, 37–41); craniofacial, skull base, and cervical spine reconstruction in neurosurgery (35); prefabrication of bony fixation plates and planning excision of bony lesions in orthopedic surgery (42, 43); mapping complex congenital heart defects and tracheobronchial variation in cardiothoracic surgery and cardiac transplantation (26, 44–52) (Figure 2); endovascular repair of abdominal aortic aneurysm and aortic dissection in vascular surgery (53–55); partial nephrectomy for renal tumors in urology (56); osteoplastic flap reconstruction of frontal sinus defects in ear, nose, and throat surgery (57, 58); and hepatectomy and liver transplantation in general surgery (59–61).

### Intraoperative guidance

Furthermore, 3D softwares have been used to fabricate patient-specific surgical templates and intraoperative guidance devices to aid surgeons in maxillofacial surgery (62–67), neurosurgery (68), orthopedic surgery (69), hand surgery (70), and general surgery (71).

### Education

3D-printed haptic biomodels can be useful for educating patients during medical consultations and training surgical trainees (29, 45, 72–81).

### Customized prosthesis

Moreover, 3D printing has enabled rapid and convenient production of customized implants. Investigators have manufactured patient-specific mandibular implants in maxillofacial surgery (82–84), cranial vault implants for cranioplasty in neurosurgery (85, 86), hip implants in orthopedic surgery (87, 88), and a bioresorbable airway splint for complex tracheobronchomalacia in pediatric cardiothoracic surgery (89).

### Allied health

In other areas of medicine, 3D printing has revolutionized the manufacturing of hearing aids and currently 99% of all hearing aids in the world are 3D printed (90). Additionally, 3D printing has helped in making complex diagnoses in forensic medicine (91); reformed anatomy education (92); helped in planning repairs of Charcot’s foot in podiatry (93); permitted the fabrication of custom-made dental implants in dentistry (94–96); produced patient-specific 3D-printed medication in pharmaceutical industry (97, 98); and assembled custom-design tissue scaffolds in regenerative medicine (99, 100).

## 3D Printing at the Bedside

Despite a vast potential scope of 3D printing in clinical practice and significant media interest with frequent reports of the latest innovative advancements made using this technology (101). The incorporation of 3D printing as a clinical bedside application has not been widespread (102). One potential barrier is the perception amongst clinicians that 3D printing is technically sophisticated and is reserved for planning intricate operations and devising highly specialized implants (102). As a result, 3D printing is often outsourced to an external company, which compounds the cost and time. This demonstrates a lack of awareness of the increasing accessibility of the 3D softwares and the declining cost of the 3D printers (102).

### 3D reconstruction software

In order to fabricate a 3D biomodel, two types of software are required; firstly, a “3D modeling” software that translates the DICOM (digital imaging and communications in medicine) files from CT/MRI scans into a CAD file, and secondly, a “3D slicing” software that divides the CAD file into thin data slices suitable for 3D printing (103).

#### 3D Modeling Software

A range of 3D modeling softwares is available (Table 2); however, early ones, such as Mimics (Materialise NV, Leuven, Belgium), would incur a high cost for the initial purchase and for the ongoing software updates. Driven by the consumerization of 3D printing and an increasing number of both professional and community software developers, free open-source softwares, such as Osirix (104) and 3D Slicer (105–107), have become widely utilized. Our group prefers using them due to the latter’s expansive developer community base, called the Slicer Community, a plethora of plug-in functions, and a user interface that is intuitive to an individual with no engineering background (108, 109). An ideal 3D modeling software should be free; capable of highlighting the region of interest and eliminate undesired areas using the threshold and the segmentation function, respectively; export the 3D model as a CAD file in a universally accepted 3D file format, such as STL (standard tessellation language); and possess an easy-to-use interface. Encouragingly, there are numerous 3D modeling softwares available in the market currently that fit all of the criteria (Table 2).

**Table 2:** **A summary of 3D modeling softwares that can convert a DICOM data from a standard CT/MRI scans into a CAD file**.

Name	Company	Free	Threshold/segmentation	Export STL	Easy user interface	OS platform
3D Slicer	Surgical Planning Laboratory	Y	Y	Y	Y	W, M
MITK	German Cancer Research Centre	Y	Y	Y	Y	W, M
Osirix	Pixmeo	Y	Y	Y	Y	M
MIPAV	NIH CIT	Y	Y	Y	N	W, M
MeVisLab	MeVis Medical Solutions AG	Y	Y	Y	N	W, M
InVesalius	CTI	Y	Y	Y	N	W, M
Mimics	Materialise NV	N	Y	Y	Y	W, M
Avizo/Amira	FEI Visualization Science Group	N	Y	Y	Y	W, M
3D Doctor	Able Software	N	Y	Y	Y	W
Dolphin Imaging 3D	Dolphin Imaging and Management	N	Y	Y	Y	W
Analyze	AnalyzeDirect	N	Y	Y	N	W, M
GuideMia	GuideMia	N	Y	Y	N	W, M
OnDemand3D	CyberMed	N	N	Y	N	W, M
VoXim	IVS Technology	N	Y	Y	N	W
ScanIP	Simpleware	N	Y	Y	N	W

*STL, standard tessellation language; OS, operating system; Y, yes; N, no; W, Windows OS; M, Mac OS*.

#### 3D Slicing Software

3D slicing softwares digitally “slice” a CAD file into layers suitable for 3D printing. However, they are also useful for altering the orientation of the CAD file relative to the printer build plate to give an optimal direction, which minimizes the requirement for the support structures and, in turn, reduces the amount of material used and therefore also reduces the printing time. This process can be readily performed using proprietary softwares that accompany the 3D printers at no extra cost and usually possess a simple graphic user interface, such as Cube software (3D Systems) and MakerBot Desktop (MakerBot Industries).

### 3D printers

The cost of early 3D printers, consisting of mostly the SLA type described above, precluded widespread adoption of 3D printing in the initial years; however, the expiration of key patents surrounding SLA and FDM in the last decade has fueled a surge in the number of commercial developers leading to an increase in the availability and a significant reduction of the cost (Table 3). Several affordable SLA 3D printers have entered the market since then, such as Form 1+ (Formlabs, Somerville, MA, USA) and ProJet 1200 (3D Systems). However, they are capable of building only small designs (i.e., 12.5 cm × 12.5 cm × 16.5 cm) and hence, remain unsuitable for many applications. Similarly, current MJM and SLS 3D printers are generally bulky and expensive, and require specialized skills for safe handling of the hardware and its maintenance. Binder jet 3D printers are gradually being avoided due to the brittle quality of the end-products and the large size of the printer. Currently, FDM 3D printers are the preferred option as a desktop application in medicine for their affordability and practicality. The accuracy and the quality of FDM products are comparable to SLA, SLS, and binder jet (110–112). Furthermore, FDM incurs the least cost in maintenance from ongoing print materials (Table 4).

**Table 3:** **A summary of commercially available 3D printers from ten leading 3D printing companies in the world**.

Type	Name	Company	Cost (USD)	Print area (cm)	Print resolution (nm)	Printer size (cm)	Printer weight (kg)
SLA	Form 1+	Formlabs	3,999	12.5 × 12.5 × 16.5	25	30.0 × 28.0 × 45.0	8
SLA	ProJet 1200	3D Systems	4,900	4.3 × 2.7 × 15.0	30.5	22.9 × 22.9 × 35.6	9
SLA	ProJet 6000	3D Systems	200,000	25.0 × 25.0 × 25.0	50	78.7 × 73.7 × 183.0	181
SLA	ProJet 7000	3D Systems	300,000	38.0 × 38.0 × 25.0	50	98.4 × 85.4 × 183.0	272
SLA	ProX 950	3D Systems	950,000	150.0 × 75.0 × 55.0	50	220.0 × 160.0 × 226.0	1,951
MJM	Objet 24 series	Stratasys	19,900	23.4 × 19.2 × 14.9	28	82.5 × 62.0 × 59.0	93
MJM	Objet 30 series	Stratasys	40,900	29.4 × 19.2 × 14.9	28	82.5 × 62.0 × 59.0	93
MJM	ProJet 3510 series	3D Systems	69,500	29.8 × 18.5 × 20.3	16	29.5 × 47.0 × 59.5	43.4
MJM	Objet Eden	Stratasys	123,000	49.0 × 39.0 × 20.0	16	132.0 × 99.0 × 120.0	410
MJM	ProJet 5000	3D Systems	155,000	53.3 × 38.1 × 30.0	32	60.3 × 35.7 × 57.1	53.8
MJM	ProJet 5500X	3D Systems	155,000	53.3 × 38.1 × 30.0	29	80.0 × 48.0 × 78.0	115.7
MJM	Connex series	Stratasys	164,000	49.0 × 39.0 × 20.0	16	140.0 × 126.0 × 110.0	430
MJM	Objet Connex series	Stratasys	164,000	49.0 × 39.0 × 20.0	16	142.0 × 112.0 × 113.0	500
MJM	Objet 1000	Stratasys	614,000	100.0 × 80.0 × 50.0	16	280.0 × 180.0 × 180.0	1,950
SLS	sPro series	3D Systems	300,000	55.0 × 55.0 × 46.0	80	203.0 × 160.0 × 216.0	2,700
SLS	ProX series	3D Systems	500,000	38.1 × 33.0 × 45.7	100	174.4 × 122.6 × 229.5	1,360
BJT	ProJet 160	3D Systems	40,000	23.6 × 18.5 × 12.7	100	74.0 × 79.0 × 140.0	165
BJT	ProJet 260C	3D Systems	40,000	23.6 × 18.5 × 12.7	100	74.0 × 79.0 × 140.0	165
BJT	ProJet 360	3D Systems	40,000	20.3 × 25.4 × 20.3	100	122.0 × 79.0 × 140.0	179
BJT	ProJet 460 Plus	3D Systems	40,000	20.3 × 25.4 × 20.3	100	122.0 × 79.0 × 140.0	193
BJT	ProJet 4500	3D Systems	40,000	20.3 × 25.4 × 20.3	100	162.0 × 80.0 × 152.0	272
BJT	ProJet 660 Pro	3D Systems	40,000	25.4 × 38.1 × 20.3	100	188.0 × 74.0 × 145.0	340
BJT	ProJet 860 Plus	3D Systems	40,000	50.8 × 38.1 × 22.9	100	119.0 × 116.0 × 162.0	363
FDM	Huxley Duo	RepRapPro	453	13.8 × 14.0 × 9.5	12.5	26.0 × 28.0 × 28.0	4.5
FDM	Mendel	RepRapPro	586	21.0 × 19.0 × 14.0	12.5	50.0 × 46.0 × 41.0	8
FDM	Ormerod 2	RepRapPro	702	20.0 × 20.0 × 20.0	12.5	50.0 × 46.0 × 41.0	6
FDM	Tricolor Mendel	RepRapPro	863	21.0 × 19.0 × 14.0	12.5	50.0 × 46.0 × 41.0	8
FDM	Cube 3	3D Systems	999	15.3 × 15.3 × 15.3	70	33.5 × 34.3 × 24.1	7.7
FDM	Buccaneer	Pirate 3D	999	14.5 × 12.5 × 15.5	85	25.8 × 25.8 × 44.0	8
FDM	Original +	Ultimaker	1,238	21.0 × 21.0 × 20.5	20	35.7 × 34.2 × 38.8	N/A
FDM	Replicator mini	MakerBot	1,375	10.0 × 10.0 × 12.5	200	29.5 × 31.0 × 38.1	8
FDM	Creatr	Leapfrog	1,706	20.0 × 27.0 × 20.0	50	60.0 × 50.0 × 50.0	32
FDM	Replicator 2	MakerBot	1,999	28.5 × 15.3 × 15.5	100	49.0 × 42.0 × 38.0	11.5
FDM	LulzBot TAZ 4	Aleph Objects	2,195	29.8 × 27.5 × 25.0	75	668.0 × 52.0 × 51.5	11
FDM	AW3D HDL	Airwolf 3D	2,295	30.0 × 20.0 × 28.0	100	61.0 × 44.5 × 46.0	17
FDM	Creatr HS	Leapfrog	2,373	29.0 × 24.0 × 18.0	50	60.0 × 60.0 × 50.0	40
FDM	Replicator 2x	MakerBot	2,499	24.6 × 15.2 × 15.5	100	49.0 × 42.0 × 53.1	12.6
FDM	Ultimaker 2	Ultimaker	2,500	23.0 × 22.5 × 20.5	20	35.7 × 34.2 × 38.8	N/A
FDM	Replicator 5th gen	MakerBot	2,899	25.2.19.9 × 15.0	100	52.8 × 44.1 × 41.0	16
FDM	AW3D HD	Airwolf 3D	2,995	30.0 × 20.0 × 30.0	60	61.0 × 44.5 × 46.0	17
FDM	Cube Pro	3D Systems	3,129	20.0 × 23 × 27.0	100	57.8 × 59.1 × 57.8	44
FDM	AW3D HDX	Airwolf 3D	3,495	30.0 × 20.0 × 30.0	60	61.0 × 44.5 × 46.0	17
FDM	AW3D HD2X	Airwolf 3D	3,995	27.9 × 20.3 × 30.5	60	61.0 × 45.7 × 45.7	18
FDM	Creatr xl	Leapfrog	4,988	20.0 × 27.0 × 60.0	50	75.0 × 65.0 × 126.0	37
FDM	Replicator Z18	MakerBot	6,499	30.5 × 30.5 × 45.7	100	49.3 × 56.5 × 85.4	41
FDM	Xeed	Leapfrog	8,705	35.0 × 27.0 × 60.0	50	101.0 × 66.0 × 100.0	115
FDM	Mojo	Stratasys	9,900	12.7 × 12.7 × 12.7	178	63.0 × 45.0 × 53.0	27
FDM	uPrint	Stratasys	13,900	20.3 × 15.2 × 15.2	254	63.5 × 66.0 × 94.0	94
FDM	Objet Dimension series	Stratasys	40,900	25.4 × 25.4 × 30.5	178	83.8 × 73.7 × 114.3	148
FDM	Fortus series	Stratasys	184,000	91.4 × 61.0 × 91.4	127	277.2 × 168.3 × 202.7	2,869

*Where a 3D printer series is characterized, the lowest cost, largest print area, lowest print resolution, largest printer size, and greater printer weight are selected for comparison. SLA, stereolithography; MJM, multijet modeling; SLS, selective laser sintering; BJT, binder jet technique; FDM, fused deposition modeling; cm, centimeter; kg, kilograms; nm, nanometers; N/A, not available*.

**Table 4:** **A summary of average raw material cost of each 3D printing technique**.

Type of 3D printing	Average cost of print material (USD)
SLA	200 per L
MJM	300 per kg
SLS	500 per kg
BJT	100 per kg
FDM	50 per kg

*SLA, stereolithography; MJM, multijet modeling; SLS, selective laser sintering; BJT, binder jet technique; FDM, fused deposition modeling; L, liter*.

## 3D Printing in Plastic and Reconstructive Surgery

In plastic and reconstructive surgery, 3D-printed haptic biomodels can potentially play a significant role in preoperative planning, intraoperative guidance, training and teaching, and fashioning patient-specific prosthesis (Table 5).

**Table 5:** **A summary of published application of 3D printing in Plastic and Reconstructive Surgery**.

Application		Example	Reference
Preoperative planning	Soft tissue mapping	Breast reconstruction	(108)
		Ear reconstruction	(113, 114)
		Nasal reconstruction	(115)
		Mandibular soft tissue tumor resection	(116)
		“Reverse” model of ankle defect	(109)
		Sacral defect	(117)
	Vascular mapping	Internal mammary artery perforators	(118)
		DIEA perforators	(7)
	Bony mapping	Basal thumb osteoarthritis	(7)
	4D printing	Thumb movement	(119)
Intraoperative guidance		Bone reduction clamp	(70)
Surgical training		N/A	
Patient education		N/A	
Patient-specific		Craniofacial implant	(120)
prosthesis		“Ear and nose library”	(121, 122)

*DIEA, deep inferior epigastric artery; 4D, four dimensional; N/A, not available*.

### Preoperative planning: Soft tissue mapping

Perforator flap surgery is routinely performed in the reconstruction of large soft tissue defects after trauma or an oncologic resection. Preoperative planning with CTA has revolutionized the field by enabling the reconstructive surgeon to identify an ideal donor site, flap, and perforator for a free flap transfer (3, 123), facilitating a greater flap success rate and an overall improvement in the clinical outcomes (1, 2, 124). In addition to CTA, 3D biomodels can provide an additional layer of clinical information through visual and tactile examination.

**Figure 3:**
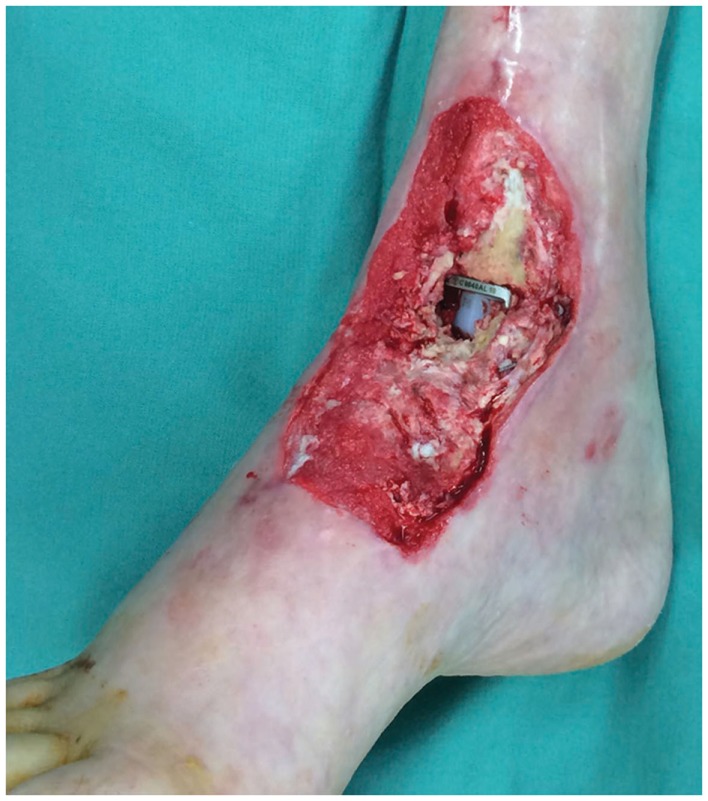
**Photograph of the soft tissue ankle defect showing the exposed metal hardware from a previous ankle reconstruction**. Reproduced with permission from *Microsurgery* (109).

**Figure 4:**
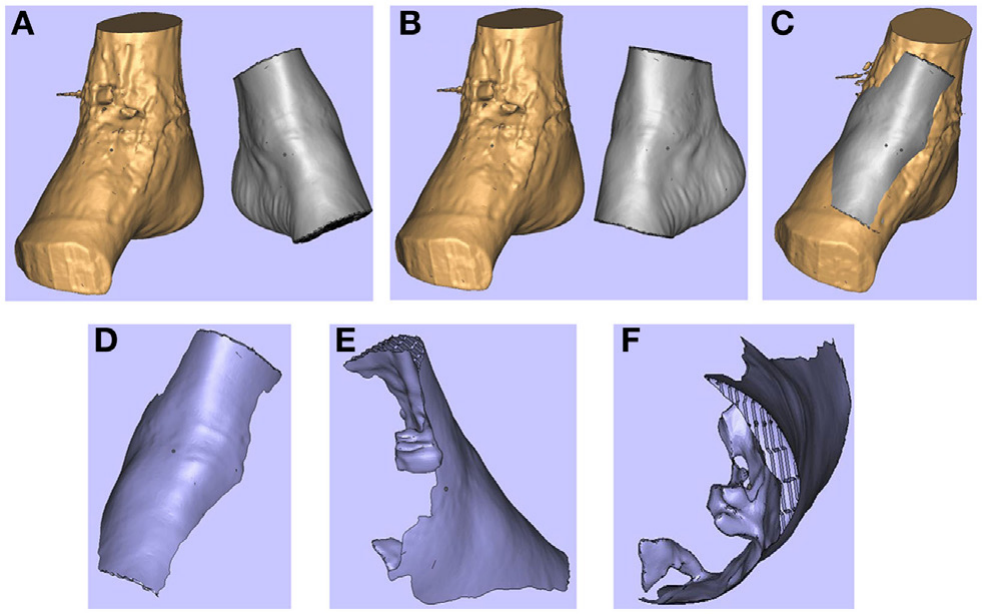
**3D images of the right (pathological) ankle is juxtaposed to the left (normal) ankle (A). The left ankle is reflected (B) and superimposed on to the right ankle (C). These images are subtracted from each other to produce a “reverse” model of the soft tissue defect (D-F)**. Reproduced with permission from *Microsurgery* (109).

**Figure 5:**
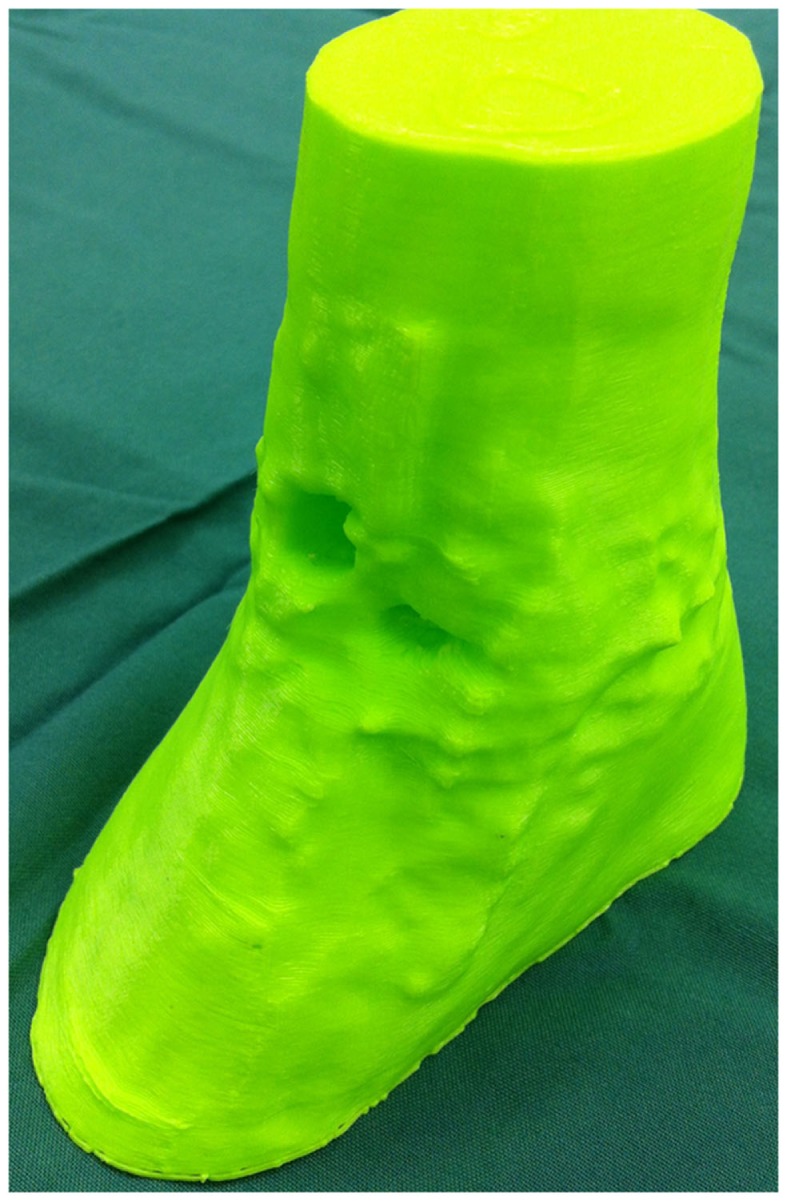
**3D-printed haptic model of the soft tissue ankle defect**. Reproduced with permission from *Microsurgery* (109).

**Figure 6:**
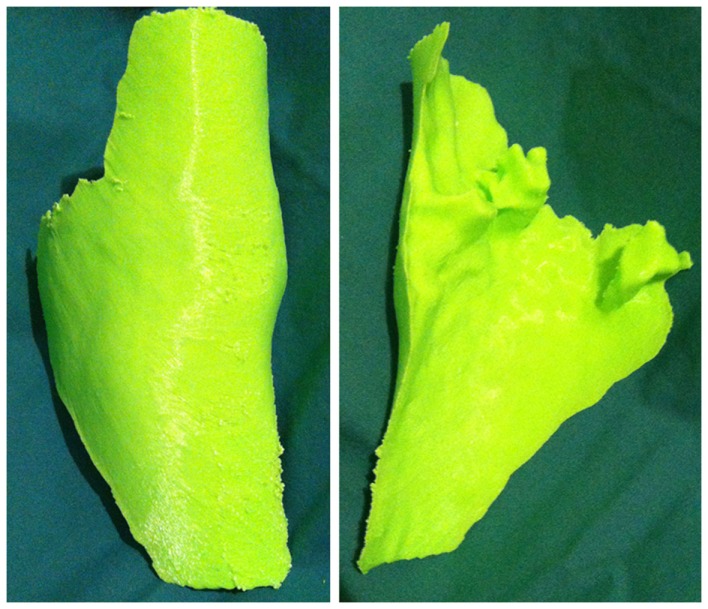
**3D-printed haptic model of the “reverse” image representing the wound defect**. Reproduced with permission from *Microsurgery* (109).

In a recent report, our research group described a technique of fashioning a “reverse” model representing a soft tissue ankle defect that was utilized for planning a perforator flap-based reconstruction (Figure 3) (109). Routine CTA of the lower limbs (i.e., recipient site) and the forearms (i.e., donor site) were conducted and the DICOM data were converted into a CAD file using Osirix. The 3D image of the normal contralateral ankle was mirrored, superimposed over the image of the pathological side, and after digital subtraction using Magics software (Materialise NV), a “reverse” model representing the wound defect is created (Figure 4). This mirroring function can also be performed in free open-source softwares, such as Osirix and 3D Slicer. This helped the surgeon preoperatively appreciate the length, width, and depth of the free flap that needed to be harvested in order to adequately cover the defect. Both the pathological ankle and the “reverse” model were fabricated in PLA filaments using a Cube 2 printer (3D Systems) (Figures 5 and 6) (Table S1 in Supplementary Material).

We also recently demonstrated the utility of a 3D-printed biomodel for planning perforator flap reconstruction of a sacral wound defect post-oncologic resection (117). Likewise, we used Osirix to translate the preoperative sacral CTA data into a CAD file. Due to the maximal build dimensions of the Cube 2 printer (i.e., 16 cm × 16 cm × 16 cm), the 3D image of the sacral defect was scaled down using the Cube software. The haptic model still accurately represented the shape and depth of the defect and its relationship with the surrounding anatomical structures.

**Figure 7:**
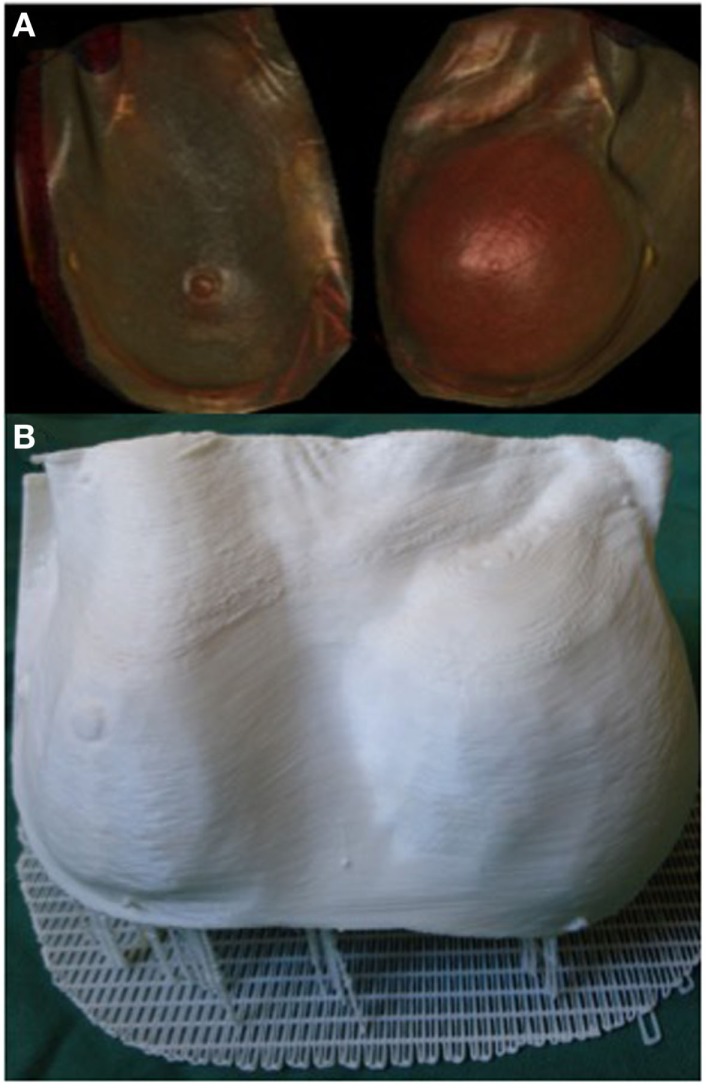
**3D reconstructed CT images of a patient with breast asymmetry post-mastectomy (A) and the 3D printed breast model of the same patient (B)**. Reproduced with permission from *Breast Cancer Research and Treatment* (108).

3D printing can potentially be a valuable tool in the assessment of soft tissue volume. Volumetric analysis is an essential component of breast reconstructive surgery and currently surgeons rely on 2D photography or 3D scanning technology, such as VECTRA (Canfield Imaging Systems, Fairfield, NJ, USA) (125), and subjective visual assessment. One of the main limitations of 3D photography like VECTRA is the inability to account for an underlying chest wall asymmetry that may incorrectly lead to an asymmetrical appearance despite equal breast parenchymal volumes. Moreover, the accuracy of each scan is reliant on the patients standing with their back flat against a wall, which may not be feasible in certain conditions, such as kyphosis or scoliosis. Recently, we reported the use of a 3D-printed model of a patient with post-mastectomy breast asymmetry for preoperative planning (Figure 7) (108). Despite being scaled down to fit the build size of the printer, having an accurate physical replica helped surgeons appreciate the difference in the breast shape and volume. Furthermore, using the segmentation function in Osirix we were able to quantify the breast parenchymal volume difference.

### Preoperative planning: Vascular mapping

Understanding the vascular anatomy of perforators and their relationship with the regional anatomical structures is critical in perforator flap surgery and to this effect, CTA is currently the gold standard preoperative investigation (1, 2, 123, 126). Recently, Gillis and Morris reported a cadaveric study where a model of internal mammary artery perforators and the neighboring ribs was fabricated using a binder jet 3D printer (ProJet x60 series, 3D Systems) (118). The authors demonstrated the benefits of physically interacting with the model and the ability to visualize it in multiple planes to aid dissection and identification of the dominant perforator. However, they also noted a significant cost associated with outsourcing the 3D printing (USD 400–1,200) and the print material was too delicate for small-size blood vessels that required post-production strengthening with wax coating.

Likewise, our group 3D printed the perforator anatomy for planning a deep inferior epigastric artery perforator (DIEP) flap breast reconstruction. From the preoperative CTA, we created a CAD file of the deep inferior epigastric artery (DIEA) with the surrounding bony landmarks using 3D Slicer and the Cube 2 printer. Despite having to scale down the model to fit the printer dimensions, surgeons could intuitively discern the arterial anatomy from the replica. Interestingly, the current technique impeded the perforators of DIEA to be 3D printed. Considering that the DICOM data of the CTA and the Cube 2 printer have a resolution of 0.625 and 0.200 mm, respectively, and the mean diameter of a DIEA perforator ranges between 1 and 1.4 mm (127), this may be most likely explained as a limitation of the 3D modeling software, 3D Slicer. This may be prevented in the future by installing free add-on software functions, such as Vascular Modeling Toolkit (VMTK, Orobix, Bergamo, Italy) in 3D Slicer, that are designed to specifically segment vascular structures. Currently, these are still early in the development phase and are difficult to manipulate without significant computer engineering proficiencies. As the field advances, we would naturally expect the user interface of these softwares to become easier to use.

### Preoperative planning: Bony mapping

3D printing bony pathology in the forearm, wrist, and hand is another suitable utility of this technology in plastic and reconstructive surgery. CT scans have been the most commonly used imaging modality for medical 3D printing. Since they readily differentiate bones, 3D printing bony structures has become well established in various surgical disciplines, such as maxillofacial surgery (20, 21, 33, 128–130), neurosurgery (35, 68, 86), and orthopedic surgery (131–135). Using Osirix and Cube 2 printer, our research group 3D printed a model of a subluxed first carpometacarpal joint. Being able to visualize the model from various angles and the tactile feedback facilitated an intuitive understanding of the anatomical relationship between the first metacarpal and the trapezium. The information was useful for planning the optimal method of reduction.

### A new evolution: 4D printing

**Figure 8:**
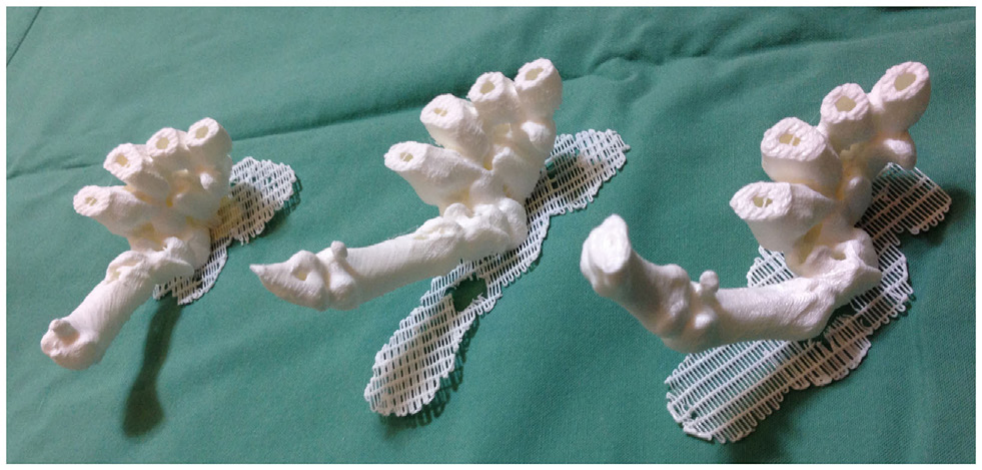
**4D-printed haptic models of carpal and metacarpal bones demonstrating thumb abduction (from left to right)**. Reproduced with permission from *Journal of Reconstructive Microsurgery* (119).

Recently, we described for the first time the concept of applying 3D printing to 4D CT scans, or 4D printing, where time is added as the fourth dimension to the standard 3D printing (119). 4D CT is a novel imaging modality developed to remove motion artifacts from organs, such as lungs, in order to enhance the image quality and facilitate precise delivery of radiotherapy (136, 137). In plastic surgery, investigators have utilized 4D CTA to assess the vascular territories and the dynamic flow characteristics of an individual perforator (4, 5). Using Osirix and Cube 2 printer, our group 3D printed the carpal and metacarpal bones of a patient in life-size at various stages of the thumb movement, such as thumb abduction (Figure 8). In contrast to the 3D reconstructions on a 2D computer screen and 3D models, 4D-printed haptic models accurately depicted the position of the carpal bones during each movement and enabled an instinctive appreciation of the spatiotemporal relationship between them. One of the major disadvantages was the reliance on the clinician reviewing the 4D CT data to select the scans most representative of the carpal bone transition during each movement for 3D printing. This can be overcome as 3D printers become faster thus allowing more models to be fabricated.

### Intraoperative guidance

The convenience of 3D printing has propelled an innovation in custom designs of surgical templates and equipments that help guide the surgeon intraoperatively. In the literature, investigators have demonstrated the utility of 3D printing a modified army/navy surgical retractor (71); patient-specific orthognathic templates to guide osteotomy (66) and mandibular fracture reduction device (138) in maxillofacial surgery; screw fixation guide system in spinal neurosurgery (139); and drill templates to aid surgical correction of multilevel cervical spine instability in orthopedic surgery (69). In plastic and reconstructive surgery, Fuller et al. illustrated how 3D printing can expedite the development of a custom-made bone reduction clamp design for hand fractures, in comparison to the conventional processes that can become protracted and actually be discouraging to innovation (70). The authors collaborated with an engineer to produce 3D prototype designs and converted them into CAD files using free 3D softwares, such as SketchUp (Trimble Navigation, Sunnyvale, CA, USA) and MeshLab (ISTI-CNR, Pisa, Italy), respectively. 3D printing of the FDM prototypes was outsourced, costing USD 75 and 1–3 days for the delivery to arrive. The final design was manufactured in metal using an additive manufacturing technique, called direct metal laser sintering, and was again outsourced, costing USD 1,200 and 2 days for the delivery. The authors acknowledged that the 3D softwares for designing prototypes are currently not intuitive for clinicians with only basic computer proficiency. Furthermore, the final cost exceeded the cost of purchasing a standard equipment. However, as 3D printing technology advances and the 3D printing is performed “in-house”, the difference may become minimal in the future.

### Surgical training

Detailed knowledge of anatomical structures and their spatial relationships are essential assets of a plastic surgeon and objectives of a surgical training program. Through the standard medical training, a surgical aspirant can gain procedural experiences from performing dissections on human cadavers as a medical student and assisting senior surgeons in the operating theater as a resident, leading toward a gradual acquisition of competence. However, human cadavers are becoming relatively scarce from the anatomical education curricula due to high maintenance costs, cultural and social controversies, and safety issues associated with the formalin-containing embalming fluids (92, 140). Furthermore, the operative experience gained as an assistant to a senior surgeon is secondary to a primary operator experience. To this end, 3D-printed anatomical models can serve as an accurate, tactile visualization tool and a surgical simulation device. Moreover, 3D-printed haptic biomodels can be utilized to reproduce complex, patient-unique pathologies that facilitate the surgical trainees to preoperatively predict potential intraoperative challenges and postoperative outcomes and aid in their learning. Subsequent improvement in the surgeon’s competence may lead to enhanced clinical outcomes and a reduced risk of complications. Investigators from various surgical disciplines have demonstrated the utility of 3D printing in training, such as neurosurgery (72–77, 141, 142), cardiothoracic surgery (54, 78–80, 143–145), urology (81, 146), and general surgery (29) However, one of the major limitations currently is the ability to print in materials that closely mimic the biomechanical properties and modulus of real human tissue as well as possessing realistic colors. As more materials enter the scope of 3D printing, future 3D-printed biomodels will be able to more closely reproduce true anatomy (50, 72, 74, 79).

### Patient education

3D-printed replicas can be useful to facilitate the physician–patient interaction during a consultation with the aim of improved understanding of the intended procedure, its potential outcomes and complications and thus can form an important aspect of informed consent. Traditional CT/MRI scans are often difficult to comprehend for patients from a non-medical background. In recent times, plastic surgeons have utilized 3D scanning technology, such as VECTRA (Canfield Imaging Systems), to accurately simulate potential outcomes from a cosmetic procedure on a computer screen (125). However, studies have consistently demonstrated that visual and tactile feedback from a 3D haptic model provides a superior understanding of anatomical details compared to 2D or 3D imaging techniques (34, 58, 147).

### Patient-specific prosthesis

As modern medicine ultimately progresses toward individualized treatment approaches, customizability of 3D printing can transform the manufacturing of patient-specific prostheses to being widely accessible and affordable. In comparison to a standard implant, a custom-made one is more likely to yield superior functional and esthetic outcomes (148, 149). Typical 3D printing materials can be sterilized using chemicals, such as Food and Drug Administration approved glutaraldehyde protocols (71), steam (20), and gas (150) for intraoperative handling. In the last decade, investigators have reported 3D-printed prostheses of nose (121, 151), ears (122, 152–155), eyes (156, 157), face (158, 159), and hand (6, 160). Furthermore, an Italian research group led by De Crescenzio and Ciocca has established an “Ear and Nose Library” where CAD files of 3D scanned ears and noses of normal university students are stocked (121, 122). When patients have pathology affecting both ears or the entire nose that impedes mirroring of the normal contralateral side to reconstruct the defect, the clinicians can select the most suitable CAD file from this database to fashion a prosthesis. In plastic surgery, standard breast implants are available in different volumes, but in a limited number of shapes. To this effect, 3D-printed breast implants customized to conform to the individual variations in the chest wall anatomy and the patient’s desired breast shape and size may lead to a more esthetic and satisfactory outcome.

Most reports have indicated that 3D-printed custom prostheses provide superior esthetics in comparison to the traditional wax-based handcrafted prosthetics (152, 154, 155). Furthermore, customized implants eschew the need to intraoperatively modify and adjust associated with the standard implants, which can directly lead to improved clinical outcomes, such as a reduction in the length of surgery, reduced exposure to anesthetics, and a decreased risk of complications like infection (161, 162). Currently, one of the major drawbacks is that most custom implants are manufactured using expensive 3D printing techniques, such as MJM (157) and SLS (151, 160). In contrast, the affordable FDM 3D printers are used to fabricate negative molds for silicone or wax-based casts, which ironically increases the overall production time and cost (121, 122, 152–154, 156, 158). This is mainly because at present, only ABS and PLA filaments are available for FDM and their hard material characteristic makes them unsuitable for producing soft tissue prosthetics. However, as research and development in 3D printing continues to grow exponentially and more materials become available for FDM, we expect to be able to directly create a custom-made prosthesis affordably in the near future.

## Future and Conclusion

In the last decade, image-guided 3D-printed haptic biomodels have proven to represent a valuable adjunct to the conventional 2D imaging modalities in plastic surgery for preoperative planning, producing intraoperative guidance tools, educating surgical trainees and patients, and fashioning patient-specific implants. In the early years, the technical complexity of 3D softwares and the prohibitive cost of 3D printers restricted accessibility of 3D printing in medicine. The expiration of key 3D printing patents has fueled an exponential development in the field and a significant reduction in the cost. Ultimately, we envision that 3D printing has the potential to become ubiquitous and function as an essential clinical bedside tool for a plastic surgeon.

## Author Contributions

All authors contributed to the preparation of this manuscript. The manuscript has been seen and approved by all authors. The content of this article has not been submitted or published elsewhere.

## Conflict of Interest Statement

The authors declare that the research was conducted in the absence of any commercial or financial relationships that could be construed as a potential conflict of interest.

## Supplementary Material

The Supplementary Material for this article can be found online at http://journal.frontiersin.org/article/10.3389/fsurg.2015.00025

Table S1 **A summary of the printing time and the amount of print material used to produce the 3D printed models in plastic and reconstructive surgery mentioned in the manuscript**.

Video S1 **A video demonstrating the binder jet 3D printing technique using a ProJet x60 series printer (3D Systems, Rock Hill, SC)**. After a layer of powder is deposited, a binder material mixed with colored dye is ejected on to the powder bed to fabricate a 3D haptic model in a layer-by-layer fashion. Filmed by PGM.
