# Supplementary material for: Emerging Applications of Bedside 3D Printing in Plastic Surgery
Source: Front Surg. 2015 Jun 16;2:25. doi: 10.3389/fsurg.2015.00025 (PMC4468745; doi:10.3389/fsurg.2015.00025)
Supplement: Table S1 — A summary of the printing time and the amount of print material used to produce the 3D printed models in plastic and reconstructive surgery mentioned in the manuscript. [file table_1.pdf]

Supplementary Table

Supplementary Table 1. **A summary of the printing time and the amount of print material used to produce the 3D printed models in plastic and reconstructive surgery mentioned in the manuscript.** Abbreviations: DIEA: deep inferior epigastric artery; CMC: carpometacarpal; 4D: four dimensional)

Model	3D Print Time (hr)	Percentage of a 3D Print Cartridge used (cost in USD)
Soft tissue ankle defect	12.5	50% (27)
“Reverse” mode of ankle defect	8.5	25% (13.5)
Soft tissue sacral defect	12.5	50% (27)
Asymmetrical breasts	15	50% (27)
DIEA	42	55% (30)
Subluxed first CMC joint	10.5	20% (11)
4D printed wrist	2-7	15% (8)
